# Extending ConWIP by flexible capacity and WIP-Cap adjustment for a make-to-order multi-item multi-stage production system

**DOI:** 10.1007/s10696-024-09547-9

**Published:** 2024-06-13

**Authors:** Balwin Bokor, Klaus Altendorfer

**Affiliations:** Department of Operation Management, University of Applied Sciences Upper Austria, Wehrgrabengasse 1-3, 4400 Steyr, Austria

**Keywords:** ConWIP, Capacity setting, Stochastic demand and processes, Production planning, Simulation

## Abstract

Constant-Work-In-Process (ConWIP) is a promising production planning and control method for make-to-order production systems, exhibiting notable potential in attaining reduced tardiness alongside effective management of work in process and finished goods inventories, as demonstrated in various studies. Furthermore, several papers show that the negative effects of high demand uncertainty, which occur when applying a make-to-order approach, can be mitigated by providing flexible capacity to coordinate demand and throughput. Therefore, in this paper the workload-based ConWIP method is combined with a flexible capacity setting method, to enable a better fit between demand and throughput. To fully capitalize on the benefits of flexible capacity and enable the production system to adapt to changes in throughput potential, an adjustment of the WIP-Cap is integrated to avoid machine starvation or unused overcapacity. To evaluate the system performance, a multi-stage multi-item make-to-order flow shop production system with stochastic demand, processing and customer required lead times is simulated. The results of a broad numerical study show a high improvement potential of the extended ConWIP version in comparison to workload-based ConWIP.

## Introduction

The consequences of short customer required lead times in combination with an increased variety of products and a scarcity of storage place prompt production companies to apply a make-to-order approach with short production lead times. For production systems, short production lead times require a low work in process inventory (WIP), i.e. a low workload evaluated in standard processing time. The standard processing time is the expected required time to process the production order at a respective machine including the setup time. Even though, a low workload enables short production lead times, a too low workload leads to low utilization and subsequently to a waste of throughput potential (Little 2011). A practical approach to achieve a workload, which enables short production lead times with simultaneously high utilization, is to control the WIP. Production planning and control methods which control the WIP and measure the throughput are referred as pull-methods (Hopp and Spearman 2004). Spearman and Zazanis (1992) showed two main advantages of pull-methods over push-methods, i.e. less congestion and easier production planning and control. The pull-method Constant-Work-In-Process (ConWIP), which has been developed by Spearman et al. (1989) and Spearman et al. (1990), controls the WIP within a production system. The control is performed by applying a predefined maximum WIP, known as the WIP-Cap. In the conventional form of ConWIP, cards associated with production orders circulate between the point of order release and completion, acting as a mechanism to control the maximum WIP in the production system (Spearman et al. 1990). This control addresses the trade-off between high utilization of machines and avoidance of excessive WIP leading to long production lead times (Hopp and Spearman 2004). To perform this control, a single WIP-Cap is being used for all products, in the sense of a system parametrization (Spearman et al. 2022). This uniformity is one of ConWIP's key advantages, as it significantly reduces the requirement for extensive data management, including the maintenance of master data. However, this approach also highlights a considerable drawback: a constrained ability for load balancing. Consequently, ConWIP is primary favourable for production systems where processing time variability is comparatively low (Germs and Riezebos 2010).

To overcome this limitation, Thürer et al. (2019) associated the WIP-Cap with workload, thereby integrating different load measurements developed by Oosterman et al. (2000) into ConWIP. The results show significant performance improvements across all performance measures when the WIP-Cap is set based on workload rather than on the count of production orders. While this ConWIP extension shares certain aspects with Workload Control (WLC) systems, particularly in its adoption of the aggregated load approach during production order release (Land and Gaalman 1996), fundamental distinctions can be made. We align with the distinctions made by Thürer et al. (2019), which highlights that WLC focuses on maintaining workload stability at individual production stages or workstation, requiring continuous feedback for real-time adjustments. Conversely ConWIP takes a broader approach, assessing the entire workload of the production system. Although Thürer et al. (2019) successfully extended ConWIP, its application in pure make-to-order systems remains somewhat limited. This limitation stems from the fluctuating capacity requirements over time, i.e. days, weeks, or months, due to stochastic customer behaviours, such as variations in demand, quantity, and lead time, as well as process uncertainty. To address these fluctuations in capacity requirement, two distinct strategies are commonly employed: the levelling strategy and the chasing strategy. The levelling strategy aims to smooth out the peaks and troughs in capacity requirements by maintaining constant capacity within each period. This smoothing is typically achieved through methods like pre-production (Majid Aarabi and Sajedeh Hasanian 2014), dynamically changing delivery times (Corti et al. 2006; Hegedus and Hopp 2001) or employing targeted marketing instruments, i.e. pricing or advertising (Birge et al. 1998; Nicholson and Pullen 1971). However, within a make-to-order production environment, the levelling strategy may not always be feasible or has additional drawbacks, i.e. additional costs. Therefore, the chasing strategy, which sets the capacity based on the capacity requirement within a period, is seen as more suitable for make-to-order production systems (Deif and ElMaraghy 2014). To execute the chasing strategy a capacity flexibility is essential. This capacity flexibility is primary achieved by using flexible work models. At flexible work models, the balance of work hours regarding every employee of the organisation, is tracked by a working-time-account. Hence, overtime is added and undertime is subtracted from the working-time-account (Lusa et al. 2009). Furthermore, a capacity requirement plan is necessary. Subsequently, the capacity requirement for every period is estimated and cumulated concerning the evaluation time window of capacity planning periods. Based on the estimated cumulated capacity requirement, different methods can be applied to calculate the required capacity to be provided. Finally, the capacity is set based on the required capacity to be provided in alignment with the working-time-account as well as upper and lower limits of the realizable capacity (Altendorfer et al. 2014).

An adjustment of the provided capacity has a direct impact on the production system's throughput potential (Hopp and Spearman 2011). A higher throughput potential allows the system to handle more workload at the same utilization or production lead time, while a lower throughput potential necessitates a reduction in workload to maintain identical utilization and production lead time (Jodlbauer 2008a). Consequently, adjusting the maximum allowed workload according to the provided capacity offers the potential for enhancing the system performance. This adjustment enables the production system to respond to changes in throughput potential and better utilize the flexible capacity. Performance is evaluated based on various cost components, including WIP costs, finished goods inventory (FGI) costs, and tardiness costs, or through production key-performance-indicators (KPIs) such as production lead time, FGI lead time and tardiness. Note that these mentioned costs and production KPIs will be further analysed in this publication.

This publication aims to build upon the foundation of workload-based ConWIP, as initially conceptualized by Thürer et al. (2019), by integrating flexible capacity and WIP-Cap adjustment. To achieve this, we will adopt and implement various capacity setting methods developed by Altendorfer et al. (2014) within the framework of a workload-based ConWIP. The capacity setting methods periodically adjust the realized capacity based on the expected workload considering limits of the realizable capacity as well as the actual state of the working-time-account. As further extension, a WIP-Cap adjustment method is developed based on the actual provided capacity. An agent-based discrete-event simulation is applied to evaluate the performance of the ConWIP extension for different planned utilization scenarios. In a numerical study, the workload-based ConWIP is compared to the capacity and WIP-Cap adjusted extended ConWIP production planning and control method. The extended ConWIP leads to scientific contributions as well as economic contributions. The scientific contributions are (1) An extended version of ConWIP, which better handles stochastic customer and shop floor behaviour based on flexible capacity and (2) An extended version of ConWIP, which continuously updates the WIP-Cap according to the provided capacity. From a managerial point of view, the performance of the ConWIP extension is observed, i.e. (A) Overall costs and (B) Production KPIs are examined. The observed overall costs are defined as the sum of WIP costs, FGI costs and tardiness costs. Capacity costs are neglected because flexible work models are applied to adjust the capacity. Thus, overcapacity can only be provided if the working-time-account has a positive time credit. In detail, the following research questions are addressed:

*RQ1:* *How* *can* *workload-based* *ConWIP* *conceptualized* *by* Thürer et al. (2019)*,* *be* *extended* *by* *flexible* *provided* *capacity* *based* *on* *current* *workload* *information* *from* Altendorfer et al. (2014) *and* *what* *is* *the* *respective* *system* *performance* *gain?*

*RQ2:* *How* *can* *the* *WIP-Cap* *be* *adjusted* *efficiently* *according* *to* *the* *flexible* *capacity* *provided* *and* *what* *is* *the* *respective* *system* *performance* *gain* *with* *the* *developed* *method?*

*RQ3:* *How* *can* *the* *capacity* *timing* *aspect* *of* *the* *applied* *flexible* *capacity* *method* *be* *improved* *to* *better* *fit* *for* *its* *ConWIP* *application* *and* *what* *is* *the* *respective* *system* *performance* *gain* *with* *the* *developed* *method?*

This publication is structured as follows. In Sect. 2 we provide a review of the relevant literature and discuss the basic mechanism of ConWIP. In Sect. 3 we develop and integrate our extensions in a workload-based ConWIP. Afterwards, the numerical study is explained in Sect. 4. Followed by the discussion of the results in Sect. 5. At the end of the publication the conclusions and further research are provided.

## State of the art

This publication makes contributions concerning the research area ConWIP as well as the research area capacity setting. Therefore, both literature streams are examined.

### ConWIP

Spearman et al. (1990) developed ConWIP as a pull-method alternative to KANBAN, where they initially associated WIP and WIP-Cap with production cards, thus positioning ConWIP within the card-based production systems category. Thürer et al. (2016) described the card-based mechanism of ConWIP and the distinction to other card-based production planning and control methods.

In detailing the operational specifics of ConWIP, production orders are sorted according to Earliest-Due-Date (EDD) and production release is only allowed if the respective due date is within the Work-Ahead-Window, which can be seen as scheduling window. A further constraint for order release is, that the WIP on the shop floor has to be smaller than the WIP-Cap. Therefore, a production order is released to the shop floor if the production order due date is smaller/equal than the Work-Ahead-Window plus the actual date and if the WIP after release is smaller/equal than the WIP-Cap (Spearman et al. 1990). The parameter Work-Ahead-Window prevents the release of production orders with a due date in the distant future, which leads to a controlled FGI (Altendorfer and Jodlbauer 2011). A control of the FGI was also explored in a recent study by Haeussler et al. (2023), in which both dynamic and constant time policies were examined within the context of workload controlled systems to strike a balance between premature completion and tardiness. Moreover, an essential aspect in production system planning, highlighted by Schneckenreither et al. (2021), is the dynamic setting of planned lead times using an artificial neural network to better reflect system dynamics and manage cost ratios between inventory holding and backorder costs. This innovative approach, tested in a high variability make-to-order flow-shop, has shown to outperform traditional forecast-based order release models in both forecast accuracy and cost efficiency. After order release, the dispatching of the production orders is originally First-In-System-First-Out (FISFO), however system performance can be enhanced by adopting alternative dispatching rules, as demonstrated by Framinan et al. (2000).

Jodlbauer and Huber (2008) recommended to measure the WIP and WIP-Cap in standard processing time, thus associating them with workload rather than with production orders. This measurement strategy ensures that the WIP-Cap determines the maximum allowable workload on the shop floor, which in turn constrains utilization and influences production lead time through Little's Law (Jodlbauer 2005; Little 1961, 2011). Thürer et al. (2019) found that linking workload with WIP-Cap leads to improvements across all performance measures, as they investigated different load measurements developed by Oosterman et al. (2000).

In the last decades, ConWIP has been a great deal of interest within the research community. Framinan et al. (2003) conducted one of the first literature reviews concerning the issues implementation decisions, application and comparison of ConWIP with other production planning and control methods. The authors determined the lack of publications which address the impact of manufacturing conditions on the system performance of ConWIP. Therefore, Jodlbauer and Huber (2008) executed a simulation study to discuss the influence of environmental robustness, which implies changing manufacturing conditions, i. e. machine reliability, scrap rate or deviation in the processing time, and parameter stability on the service level. The results imply a low robustness as well as low stability of ConWIP. Consequently, ConWIP requires an exact parameterization and stable manufacturing conditions to achieve a high service level.

The main parameter which influence the performance of ConWIP is the WIP-Cap (Framinan et al. 2006). Framinan et al. (2003) distinguished between WIP-Cap setting, i.e. determining a fixed WIP-Cap under specific manufacturing conditions to achieve acceptable system performance, and WIP-Cap controlling, i.e. creating rules to adjust the WIP-Cap in response to manufacturing events for targeted performance. Jodlbauer (2008b) developed a method to set the WIP-Cap based on a continues approach of Littles Law published by Jodlbauer and Stöcher (2006). However, Framinan et al. (2006) demonstrated that for a make-to-order production system, controlling the WIP-Cap proves to be more effective than merely setting it, regardless of their applied method. An analytical approach for controlling the WIP-Cap through threshold values involves adjusting the WIP-Cap if throughput or service levels fall below or exceed specific thresholds. This method is exemplified by the Statistical Throughput Control (STC) proposed Hopp and Roof (1998) or the dynamic card controlling technique by Framinan et al. (2006). Using the STC method, the card count will converge to a minimum level, which reaches the target throughput. Moreover, the method shows the connection between throughput potential and optimum WIP-Cap. An optimization approach to control the WIP-Cap has been conducted by Belisario et al. (2015), aiming to minimize both the overall costs and the frequency of WIP-Cap adjustments. They analysed the trade of between these two objectives by establishing the Pareto front through varying weighting factors. Whereas, Azouz and Pierreval (2019) controlled the WIP-Cap based on a multilayer perceptron neural network (MLP-NN). To set the neural network weight they did not use training data, instead they applied simulation-based optimization, which they solved heuristically by NSGA-II. Regardless of the applied WIP-Cap control method setting the parameters, i.e. thresholds or weights, is essential as they significantly influence system performance (Gonzalez-R et al. 2011).

By adapting the WIP-Cap a modified or hybrid ConWIP production planning and control method emerges. Prakash and Chin (2014) presented a literature review as well a classification method of modified ConWIP versions. The authors aggregated the publications to 15 modified ConWIP versions. The most common modifications are the implementation of several ConWIP loops followed by the combination of ConWIP with other production planning and control methods and different card setting methods.

The system performance of ConWIP have been investigated in numerous publications as in Koh and Bulfin (2004), Khojasteh-Ghamari (2009) or Li. (2011). Bertolini et al. (2015) compared the performance of ConWIP and WLC against a standard push system, using a simulation-based approach. The results revealed a trade-off, with ConWIP and WLC reducing WIP and production lead times but at the cost of lower throughput, as production orders must wait in a pre-shop pool. Moreover, their findings underscored the need for more advanced simulation models and highlighted areas requiring further investigation. Jaegler et al. (2018) summarized in a literature review the system performance of ConWIP compared to other production planning and control methods, the implementation environment of ConWIP as well as the used approaches in the publications. The authors concluded that the system performance depends on structural parameters, such as shop-type, product mix or routing requirements. Therefore, decision makers have to select one of the numerous publications, which reflects their structural parameters. Moreover, the authors determined that most publications focus on ConWIP in a make-to-order environment and simulation is by far the most frequently used research approach.

Recently, Spearman et al. (2022) provided a comprehensive account of the origins of ConWIP and addressed numerous issues and misunderstandings that have arisen over the past three decades.

### Capacity setting

Capacity setting describes the capacity decision on an operative level within the traditional hierarchical Manufacturing resource planning (MRPII) (Hopp and Spearman 2011). Thus, the capacity setting decides about the provided capacity in the near future (e.g., the provided capacity within the next week), regarding every resource in the production system. The basis, for the capacity setting, is the estimation of the capacity requirement.

One of the first capacity adjustment problems have been examined in Manne (1961), Luss (1982), Segerstedt (1996) or Kok (2000). Manne (1961) determined the capacity requirement by including probabilities in the demand rate and considering backlogs. Afterwards, whenever the capacity requirement exceeded the normal capacity the capacity is increased. Luss (1982) conducted a literature review to unify the existing literature on capacity setting problems, covering a broad spectrum of applications, i. e. process industries or water resource systems. Segerstedt (1996) implemented a cumulated capacity concept as a constraint in a numerical model for a multi-stage inventory and production control problem. The numerical approach minimizes inventory costs and shortage cost, whereby the cumulated capacity requirement has to be lower or equal the cumulated provided capacity within a period. Kok (2000) compared a fixed capacity approach, where demands are exceeded if the capacity requirement exceeds the available capacity, with a flexible adapting capacity achieved through hiring personnel.

Buitenhek et al. (2002) designed an algorithm to determine the maximum production rate for Flexible Manufacturing Systems with various part types and fixed ratios. Specifically designed to accommodate the complexities of various part types and dedicated resources, their algorithm can be applied to assess whether a particular manufacturing system has the capacity to achieve the desired throughput. Whereas, Jodlbauer (2008b) developed an analytic model to determine make-to-order capability. The make-to-order capability depends on the provided capacity, the customer required lead time distribution and the demand uncertainty. Another approach is to observe the trade-off between capacity costs and capital employed costs as in Jodlbauer and Altendorfer (2010). The authors conducted a numerical study to demonstrate the relationship between available capacity and inventory needed to achieve a predefined service level for a multi-product make-to-order production system. The results imply that capacity flexibility reduces overall costs, and the double of the surplus inventory cost has to be equal to the excess capacity cost in order to ensure minimum overall cost.

Other publications observed the behaviour of queuing system when switching between different capacity levels as in Mincsovics and Dellaert (2009) or Buyukkaramikli et al. (2013). In the publication of Mincsovics and Dellaert (2009) an analytic approach to determine an up-switching and down-switching capacity point for a continuous setting is developed. Their analytic approach considers capacity costs, capacity switching costs and lost sales costs. Buyukkaramikli et al. (2013) examined a periodical capacity setting approach with two possible capacity levels. The authors developed a search algorithm to determine the capacity levels and the switching point that minimizes the capacity-related costs.

Recent advancements in capacity setting have been addressed by Thürer and Stevenson (2020) as well as Cheng et al. (2022). Thürer and Stevenson (2020) examined forward and backward finite loading techniques, along with a workload trigger method, within a make-to-order context. While all methods exhibited performance enhancements, they underscored the prominence of the workload trigger method, which outperformed the others. On a different note, Cheng et al. (2022) developed two computationally efficient approximate dynamic programming approaches for capacity planning in a multi-factory, multi-product supply chain under demand uncertainties. Their approaches were evaluated using a real-world business case, demonstrating a simpler implementation and a higher efficiency compared to Stochastic Dynamic Programming.

Altendorfer et al. (2014) used the cumulated capacity approach of Segerstedt (1996) as well as the capacity adjustment models of Mincsovics and Dellaert (2009) and Buyukkaramikli et al. (2013) to develop different periodical capacity setting methods for a multi-stage production system. The authors compared different cumulated capacity requirement estimation methods and different capacity providing methods based on service level and mean tardiness. These promising methods are in the current publication extended to be linked with ConWIP.

## Model development

To address the issue of fluctuating capacity requirements in a make-to-order production system, the workload-based ConWIP conceptualized by Thürer et al. (2019), is extended in this paper by the system load dependent capacity setting method developed in (Altendorfer et al. 2014). As highlighted above, a dynamic WIP-Cap based on the actual realized capacity is introduced as second extension to ensure the maximum benefit from the flexible capacity.

### Workload-based ConWIP

A workload-based ConWIP multi-item multi-stage production system, where WIP and WIP-Cap is measured in standard processing time, is investigated as conceptualized by Thürer et al. (2019). Production orders are sorted based on EDD and order release takes place if the due date is smaller/equal than the Work-Ahead-Window plus the actual date and if the WIP after release is smaller/equal than the WIP-Cap (Spearman et al. 1990)*.* At completion of the production order (after the last machine), the WIP is reduced by the workload of the completed production order, which is the sum of the standard processing time regarding all required machines. The finished goods remain in the FGI until the customer required due date is reached.

### ConWIP extensions

To include the capacity adjustment method from (Altendorfer et al. 2014) and a dynamic WIP-Cap, the workload-based ConWIP is extended as follows. We study a flow shop production system with four machines and calculate the capacity requirement for each machine to decide the respective capacity provided for the whole production system. We assume that capacity can only be adjusted for the whole production system in the sense of increasing or decreasing the provided capacity of the system. Furthermore, we assume that flexible capacity is based on a working-time-account and no overtime is accepted. In detail, a decrease in provided capacity leads to an increase in working-time-account and only if the working-time-account is positive also an increase in provided capacity is possible. These assumptions concerning flexible capacity are consistent with Altendorfer et al. (2014). To enable the calculation of capacity requirement, a capacity due date is calculated for each machine based on a backward scheduling algorithm for each production order. The capacity and WIP-Cap adjustment are performed at every repeat period *∆* according to the following steps (details see in the following subsections):

1. *Estimate* *the* *capacity* *requirement* for each machine based on the currently known production orders on the shop floor and in the release list which have a due date within the Capacity Horizon, i.e. only production orders with a due date within the Capacity Horizon are considered. Note that the capacity due dates and the standard processing times of orders are applied to create a cumulative capacity requirement curve.
2. *Calculate the capacity to be provided* within the Capacity Horizon for each machine without considering any constraints and take the maximum capacity to be provided over all machines. This is the *unbounded* *capacity* *to* *be* *provided*.
3. *Calculate* *the* *realized* *capacity* *to* *be* *provided* for the next period based on the unbounded capacity to be provided taking into account upper and lower limits of realizable capacity within a repeat period and the actual state of the working-time-account.
4. *Calculate* *the* *new* *WIP-Cap* based on the realized capacity to be provided.
5. *Fix* *capacity* *plus* *WIP-Cap* for one repeat period *∆* and *update* *the* *working-time-account*.

For several steps, different approaches are implemented and tested, the following subsections show the details.

#### Estimate the capacity requirement

To estimate the capacity requirement, a capacity due date *CD*_*m,i*_. for each production order *i* at each machine *m* is calculated based on the customer order due date *D*_*i*_. Note that *M* is the number of machines, the first machine is *m* = 1 and succeeding machines have increasing *m*. *I* is the set of orders with due date within the Capacity Horizon *δ* and *t*_*c*_ is the current (simulation) time. Equation (1) shows the calculation of capacity due dates according to Altendorfer et al. (2014) where the latest possible capacity due dates, i.e. without any buffer, are applied.

1 $$CD_{m,i} = D_{i} - \sum\limits_{k = m + 1}^{M} {E\left[ {P_{k,i} } \right]} \;\quad \forall m\; \in \;M,\forall i\; \in \;I$$

In Eq. (1), the backward scheduling within the capacity due date only includes the standard processing time of the succeeding machines but no buffer. To include some buffer, Altendorfer et al. (2014) applied a statistical capacity due date buffer calculation method based on the customer required lead time distribution to push up the capacity due date. As other simulation studies, e.g. van Kampen et al. (2010) show that also a simple safety lead time improves the system performance (higher service level and lower tardiness) in case of stochastic customer behaviours, we implement such a fixed safety lead time to better fit the method to ConWIP and keep this ConWIP extension practically traceable. The following equation shows how such a safety lead time *S* can be applied to calculate the production order due date *O*_*i*_ based on the customer order due date *D*_*i*_.

2 $$O_{i} = D_{i} - S$$

Applying this to Eq. (1) leads to the capacity due date including a buffer:

3 $$CD_{m,i} \left( {P_{ \cdot ,i} } \right) = O_{i} - \sum\limits_{k = m + 1}^{M} {E\left[ {P_{k,i} } \right]} \quad \forall m\; \in \;M,\forall i\; \in \;I$$

The approach from Eq. (3) is denoted as *processing* *time-based* *backward* *scheduling*. Note that for *S* = 0, this is still equal to the approach of Altendorfer et al. (2014), however, the safety lead time is an extension. Since this $CD_{m,i} \left( {P_{ \cdot ,i} } \right)$ from Eq. (3) still includes no buffer between the single machines but only at the last machine, in addition to Altendorfer et al. (2014), a second extension with equal buffer based backward scheduling is implemented as follows:

4 $$CD_{m,i} \left( {OR_{i} } \right) = O_{i} - \left( {\left( {\frac{{O_{i} - OR_{i} }}{M}} \right)\left( {M - m} \right)} \right)\;\quad \forall m\; \in \;M,\forall i\; \in \;I$$

This Eq. (4) shows the calculation of *capacity* *due* *dates* *with* *equal* *buffers* $CD_{m,i} \left( {OR_{i} } \right)$ for each processing step and a safety lead time. Based on these capacity due dates and the standard processing time *P*_*m,i*_ the cumulative capacity requirement $A_{m} \left( {t,E\left[ {P_{m, \cdot } } \right]} \right)$ for each time *t* can be calculated as follows:

5 $$A_{m} \left( {t,E\left[ {P_{m, \cdot } } \right]} \right) = \sum\limits_{{\left( {\left. i \right|CD_{m,i} \le t} \right)}} {E\left[ {P_{m,i} } \right]} \quad \forall m\; \in \;M$$

**Fig. 1:**
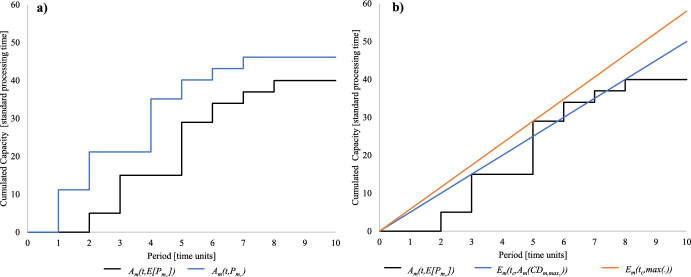
**a** Estimated capacity requirement, **b** Unbounded capacity to be provided

This cumulative capacity requirement from Eq. (5) is denoted as *standard* *processing* *time* *based* *cumulative* *capacity* *requirement* $A_{m} \left( {t,E\left[ {P_{m,\cdot} } \right]} \right)$. Since this cumulative capacity requirement does not include processing time uncertainties, Altendorfer et al. (2014) introduce a statistical method to estimate the *uncertainty* *based* *cumulative* *capacity* *requirement* $A_{m} \left( {t,P_{m, \cdot } } \right)$ as follows. Note that safety value *β* specifies the level of overcapacity included in the estimation.

6 $$A_{m} \left( {t,P_{m, \cdot } } \right) = F^{ - 1}_{{\sum\limits_{{\left( {\left. i \right|CD_{m,i} \le t} \right)}} {P_{m,i} } }} \left( \beta \right)\quad \forall m\; \in \;M$$

whereby $F^{ - 1}_{{\sum\limits_{{\left( {\left. i \right|CD_{m,i} \le t} \right)}} {P_{m,i} } }} \left( \beta \right)$ is the inverse of the CDF (cumulative distribution function) of the cumulated capacity requirement until time *t* with probability *β*. Figure 1a illustrates the calculation of $A_{m} \left( {t,E\left[ {P_{m, \cdot } } \right]} \right)$ and $A_{m} \left( {t,P_{m, \cdot } } \right)$.

#### Calculate the unbounded capacity to be provided

The calculation of the unbounded capacity to be provided is based on the estimated cumulative capacity requirement $A_{m} \left( \cdot \right)$. For the calculation two different methods, which are introduced in Altendorfer et al. (2014), are investigated.

At the *full-utilization-method*, the unbounded capacity $E_{m} \left( {t_{c} ,A_{m} \left( {CD_{m,\max } , \cdot } \right)} \right)$ for every period within the repeat period *∆*, equals the average estimated capacity requirement. Hence, the estimated capacity requirement is divided by the timespan between last considered capacity due date *CD*_*m,max*_ within the Capacity Horizon *δ* and current simulation time *t*_*c*_.

7 $$CD_{m,max } = \max \left\{ {CD_{m,i} } \right\}\quad \forall m\; \in \;M$$

8 $$E_{m} \left( {t_{c} ,A_{m} \left( {CD_{m,max } , \cdot } \right)} \right) = \frac{{A_{m} \left( {CD_{m,max } } \right)\;}}{{CD_{m,max } - t_{c} }}\quad \forall m\; \in \;M$$

The *full-utilization-method*, which is shown in Eq. (8), can be applied for $A_{m} \left( {CD_{m,max } ,E\left[ {P_{m, \cdot } } \right]} \right)$ and for $A_{m} \left( {CD_{m,max } ,P_{m, \cdot } } \right)$. It does not include safety capacity to account for uncertainties in processing time or demand. However, in case the *uncertainty* *based* *cumulative* *capacity* *requirement* $A_{m} \left( {CD_{m,max } ,P_{m, \cdot } } \right)$ is used for the calculation, the machine will have idle times, since a safety capacity is integrated in the capacity requirement estimation. To include safety capacity at the calculation of the unbounded capacity, another approach, which is denoted as *maximum-safety-method* $E_{m} \left( {t_{c} ,\max \left( \cdot \right)} \right)$*,* is tested. At the *maximum-safety-method*, the unbounded capacity per period equals the maximum slope of the estimated capacity requirement within the Capacity Horizon. As an assumption at least one production order has to be in the production system. Note that all backlog orders have a capacity requirement at current simulation time *t*_*c*_.

9 $$E_{m} \left( {t_{c} ,\max \left( \cdot \right)} \right) = \mathop {\max }\limits_{{}} \left( {\frac{{A_{m} (t)}}{{\left( {t - t_{c} } \right)}}} \right)\quad \forall m\; \in \;M$$

Figure 1b illustrates the calculation of $E_{m} \left( {t_{c} ,A_{m} \left( {CD_{m,max } , \cdot } \right)} \right)$ and $E_{m} \left( {t_{c} ,\max \left( \cdot \right)} \right)$ using the *standard* *processing* *time* *based* *cumulative* *capacity* *requirement* $A_{m} \left( {t,E\left[ {P_{m, \cdot } } \right]} \right)$.

#### Calculate the realized capacity to be provided

For the investigated flow shop production system, we assume that all machines have to provide equal capacity. From a practical point of view, this means that the machines have the same shift calendar and overtime decisions. To avoid capacity shortages, $E\left( {t_{c} } \right)$ from Eq. (10) is taken to calculate the *actual* *realized* *capacity* $C\left( {t_{c} ,E\left( {t_{c} } \right)} \right)$ for the production system. $E\left( {t_{c} } \right)$ is the maximum of the calculated unbounded capacity $E_{m} \left( {t_{c} , \cdot } \right)$ (either $E_{m} \left( {t_{c} ,A_{m} \left( {CD_{m,max } , \cdot } \right)} \right)$ or $E_{m} \left( {t_{c} ,\max \left( \cdot \right)} \right)$) for all *m* machines.

10 $$E\left( {t_{c} } \right) = \max \left( {E_{m} \left( {t_{c} , \cdot } \right)} \right)$$

However, the *actual* *realized* *capacity* also has to consider the available time on the *working-time-account* *WTA* as well as the *upper* *and* *lower* *limits* *of* *the* *realizable* *capacity* (*C*_*min*_ and *C*_*max*_) within a *repeat* *period* *∆*. Only one *working-time-account* *WTA* is used for the whole production system, which tracks the deviations from normal working times. Moreover, the *working-time-account* *WTA* is capped between zero and an *upper* *limit* *WTA*_*max*_. Therefore, an increase in *actual* *realized* *capacity* is only possible if the *working-time-account* *WTA* is positive. A decrease in *actual* *realized* *capacity* is only possible if the *working-time-account* *WTA* is below the *upper* *limit* *WTA*_*max*_. The *upper* *and* *lower* *limits* *of* *realizable* *capacity* are required, since real world companies cannot adjust the capacity by any deviation range (e. g. it is not possible to adjust the capacity by 100%). This leads to the following two cases for *actual* *realized* *capacity*:

11 $$C\left( {t_{c} ,E\left( {t_{c} } \right)} \right) = \min \left( {E\left( {t_{c} } \right),C_{max } ,WTA + C_{norm} } \right){\text{ for }}E\left( {t_{c} } \right) > C_{norm}$$

12 $$C\left( {t_{c} ,E\left( {t_{c} } \right)} \right) = \max \left( {E\left( {t_{c} , \cdot } \right),C_{min } ,C_{norm} - \left( {WTA_{max } - WTA} \right)} \right){\text{ for }}E\left( {t_{c} } \right) < C_{norm}$$

Note that based on the two options for calculating $A_{m} \left( \cdot \right)$ and the two options for calculating $E_{m} \left( \cdot \right)$, the calculation of $E\left( {t_{c} } \right)$ in Eqs. (11) and (12) can be applied for four different combinations of $A_{m} \left( \cdot \right)$ and $E_{m} \left( \cdot \right)$, all of which are tested in the simulation study. For the workload-based ConWIP without capacity flexibility, no working-time-account is available which has to be considered when the numerical results are interpreted.

#### Calculate the new WIP-Cap

The adjustment of the actual realized capacity leads to a change in the throughput potential of the production system. Hence, a different workload can be processed. However, due to the WIP-Cap, the production systems workload is limited, regardless of the actual realized capacity. As a result, two negative situations can occur. In situation one, the actual realized capacity is higher than the normal capacity, but additional production order release is not possible due to the WIP-Cap. Consequently, the higher throughput potential is not exploited, and machine idle times can occur. In situation two, the actual realized capacity is lower than the normal capacity but workload is still released into the production system due to the WIP-Cap. Because of the lower throughput potential, the production lead time as well as the utilization rises. To avoid both negative effects, the WIP-Cap is adjusted based on the actual realized capacity in this paper.

The developed approach to adjust the WIP-Cap, is to apply a certain *WIP-Cap-Change-Ratio* *R*. The *WIP-Cap-Change-Ratio* indicates the connection between capacity adjustment and WIP-Cap adjustment. The *WIP-Cap-Change-Ratio* is an additional parameter which has to be set for the ConWIP production system. Based on the *WIP-Cap-Change-Ratio*, the *determined* *WIP-Cap* *for* *normal* *provided* *capacity* *W*_*norm*_ and the realized capacity adjustment, the *adjusted* *WIP-Cap* *W*_*c*_ can be calculated as follows:

13 $$W_{c} \left( {t_{c} } \right) = W_{norm} \left( {1 + \left( {\frac{{C\left( {t_{c} , \cdot } \right) - C_{norm} }}{{C_{norm} }}} \right)R} \right)$$

**Fig. 2:**
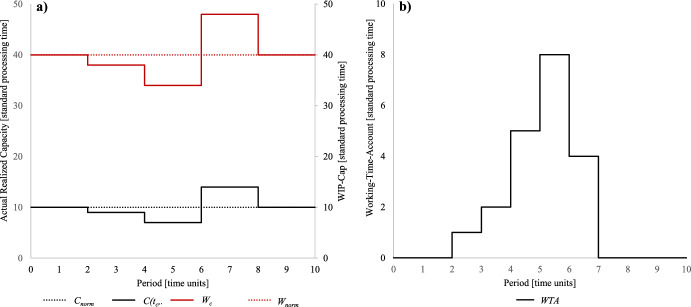
Relationship actual realized capacity, adjusted WIP-Cap and working-time-account

For clarification a short example is introduced: Assume the *normal* *realized* *capacity* *within* *a* *repeat* *period* *C*_*norm*_ is ten units of standard processing time. Moreover, the *determined* *WIP-Cap* *for* *normal* *realized* *capacity* *W*_*norm*_ is determined as 40 units of standard processing time. A *WIP-Cap-Change-Ratio* *R* of 0.5 is applied. The *actual* *realized* *capacity* $C\left( {t_{c} , \cdot } \right)$ is decreased by 10% compared to the *normal* *realized* *capacity* *C*_*norm*_. For *R* = 0.5, the capacity increase leads to a WIP-Cap decrease of 5%. Hence, in this repeat period *∆* the *actual* *realized* *capacity* $C\left( {t_{c} , \cdot } \right)$ equals nine and the *adjusted* *WIP-Cap* *W*_*c*_ = 38, both evaluated in standard processing time. Figure 2a shows the relationship between *actual* *realized* *capacity* and *adjusted* *WIP-Cap*, while Fig. 2b illustrates the development of the corresponding *working-time-account* *WTA* over ten periods with a *repeat* *period* *∆* = 2. Figure 2a shows that during the initial stages of the observation period, there is a decline in realized capacity and WIP-Cap, which leads to an increase in the working-time-account shown in Fig. 2b. As the observation period progresses, the realized capacity and WIP-Cap continue to decrease, resulting in further accumulation of positive time credit in the working-time-account. However, towards the end of the observation period, overcapacity is introduced, causing an increase in the WIP-Cap and leading to a complete utilization of the positive time credit in the working-time-account.

#### Fix capacity plus WIP-Cap and update the working-time-account

The last step is to fix the *actual* *realized* *capacity* and the *adjusted* *WIP-Cap* for the *repeat* *period* *∆.* Based on the *actual* *realized* *capacity*, the *working-time-account* *WTA* has to be updated as follows:

14 $$WTA(t_{c} ) = WTA(t_{c} - \Delta ) + \left( {C_{norm} - C\left( {t_{c} , \cdot } \right)} \right)$$

## Simulation model and numerical study

**Fig. 3:**
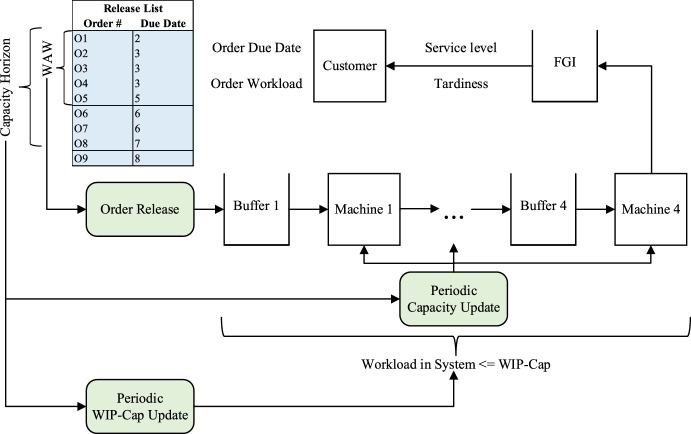
Observed production system

The numerical study is conducted for evaluating the developed ConWIP extensions at different planned utilization scenarios. A multi-stage multi-item, make-to-order flow shop production system with stochastic demand, processing, and customer required lead times is observed. The observed production system consists of four machines and is illustrated in Fig. 3. For each customer order, one production order is created for the customer specific item. The standard processing time for a production order, which is equal to the expected production time (i.e. no bias is modelled), varies for each production order at each machine. However, the mean standard processing time for all production orders is identical for each machine. Thus, no bottleneck machine exists, and the workload is on average equal for every machine. Since each production order is produced individually, the standard processing time includes setup and transportation times. A production release is only possible if the current WIP, evaluated in standard processing time, plus the workload of the production order, which is the sum of the standard processing time regarding the four machines, is below the WIP-Cap. Production orders are sorted for release according to the EDD rule. In case of a production release, the WIP is increased by the workload. The dispatching of the production orders within the production system is FISFO. Since it is a flow shop production system, a FISFO dispatching equals a First-In-First-Out (FIFO) dispatching rule. At completion of the production order (after the last machine), the WIP is reduced by the workload of the completed production order. The finished goods remain in the FGI until the customer required due date is reached. In case of tardiness, the delivery is executed immediately.

To evaluate the system performance sensibility related to utilization, four different planned utilization scenarios ∈ {0.82, 0.86, 0.90, 0.94} are simulated. The planned utilization is adapted by changing the inter-arrival time of customer orders, which is lognormal distributed with a coefficient of variation of 0.5 and mean values of {2.56, 2.44, 2.33, 2.23}. The customer required lead time consists of two parts: a deterministic part and a stochastic part. The deterministic part is equal to 2.5 times the total mean standard processing time for the four machines. This part guarantees a minimum required lead time of 21 periods. On the other hand, the stochastic part is twice the length of the total mean standard processing time for the four machines. This part follows a lognormal distribution with a mean of 16.8 periods and a coefficient of variation of 1. Both lengths where determined based on the results of preliminary studies. For each production order at each machine, a standard processing time is drawn from a lognormal distribution with mean 2.1 and a coefficient of variation of 0.2. This leads to different workloads of each production order which are applied for the WIP-Cap based order release and the capacity setting. Based on these standard processing times, the stochastic realized processing times are drawn from a lognormal distribution with a coefficient of variation of 0.2. Note that the stochastic realized processing time is not known in advance for production planning but leads to shop floor uncertainty. The log-normal distribution was selected due to its non-negativity and its widespread application in previous studies.

To ensure a fair comparison among different method combinations, we conduct a parameter enumeration for both workload-based ConWIP and our proposed extensions. Notably, contrasting our approach with other WIP-Cap controlled methods, as outlined in Sect. 2.1*.* *Workload-Based* *ConWIP* (including those developed by Hopp and Roof (1998), Belisario et al. (2015) or Azouz and Pierreval (2019)), does not provide a balanced evaluation due to their lack of flexible capacity utilization. This limitation is critical, as Tan and Alp (2009) have mathematically proofed the superiority of flexible over fixed capacity, especially in scenarios characterized by high demand uncertainty and extensive capacity flexibility, i.e. wide range between upper and lower limits of the realizable capacity. Therefore, our study is specifically directed to comparing the proposed extension to the workload-based ConWIP, as conceptualized by Thürer et al. (2019), because this approach has already been proven to outperform the conventional ConWIP system.

We identify and report the best-performing planning parameters concerning overall costs. For workload-based ConWIP, the planning parameters WIP-Cap, measured in standard processing time, and Work-Ahead-Window, measured in time units, are enumerated in the range *WIP-Cap* ∈ {35, 50, …, 200} and *Work-Ahead-Window* ∈ {16, 20, …, 52}. As for the extended versions of ConWIP, we perform a parameter enumeration within the ranges of *WIP-Cap* ∈ {35, 50, …, 155} and *Work-Ahead-Window* ∈ {16, 20, …, 36}. To determine the capacity due dates both the *processing* *time-based* *backward* *scheduling* approach and the *capacity* *due* *dates* *with* *equal* *buffers* approach are investigated with a safety lead time *S* ∈ {0, 6, …, 36} time units. For estimating the capacity requirement (A_m_), the *Capacity* *Horizon* is varied within {20, 26, …, 56} time units and both the *standard* *processing* *time* *based* *cumulative* *capacity* *requirement* as well as the *uncertainty* *based* *cumulative* *capacity* *requirement* are tested. Note, that parameter combinations where the *Capacity* *Horizon* is smaller than the *Work-Ahead-Window* are excluded from investigation, since all production orders which are considered for production release are also considered for capacity calculation. In the *uncertainty* *based* *cumulative* *capacity* *requirement*, the probability for the inverse of the cumulated distribution function is set to *β* = 0.9, which is determined based on preliminary studies. For calculating the unbounded capacity to be provided (*E*_*m*_) both the *full-utilization-method* and the *maximum-safety-method* are tested. The repeat period *∆* equals 24 h, since it is assumed that capacity is adapted daily. The total simulation time equals four years. However, a warm-up period of one year is excluded from the measured results. The upper limit of the working-time-account *WTA*_*max*_ is set to 1500 h. Since all strategies use the capacity flexibility based on the working-time-account and the *WTA* status at the end of the simulation run mainly depends on the last few capacity decisions, no additional costs for the status of the working time account at the end of the simulation have been included to avoid additional randomness in the simulation results. Note that the simulation runtime is rather long compared to the maximum working time account, i.e. in 4 years 140–160 h (= 4 years x 365 days x 24 hours x 4 machines) of normal capacity are provided and the maximum WTA is with 1 500 h about 1% of that. Moreover, the working-time-account *WTA* is in the beginning of the simulation run zero. The behaviour of the extended ConWIP production planning and control method is examined for two different ranges of capacity flexibility ∈ {0.1, 0.3}, effecting the upper and lower limits of realizable capacity (C_*min*_/C_*max*_). In the simulation model, capacity adjustment measures effect the machine speed as in Altendorfer et al. (2014). To adjust the WIP-Cap based on the actual realized capacity, different *WIP-Cap-Change-Ratio* *R* ∈ {0, 0.25, …, 1.5} are tested. The calculated overall costs include WIP costs {0.5 / time unit}, FGI costs {1/time unit} and backorder costs {19 / time unit}. 10 replications are generated for each parameter set and the simulation is conducted in AnyLogic 8.8.2. In this numerical study, 480 parameter sets for workload-based ConWIP and 959 616 parameter sets for the capacity and WIP-Cap adjusted extended ConWIP production planning and control method have been investigated. Table 1 presents a comprehensive overview of the numerical study, including the tested parameter sets and the investigated ranges.

**Table 1:** Overview numerical study

Parameter description	Investigated range for ConWIP	
	Workload-based	Extension
Planned utilization scenario	{0.82, 0.86, 0.94}	{0.82, 0.86, 0.94}
WIP-Cap [SPT]	{35, 50, 200}	{35, 50, 155}
Work-ahead-window [TU]	{16, 20, 52}	{16, 20, 36}
Approaches to determine capacity due dates	–	2 approaches
Safety lead time [TU]	–	{0, 6, 36}
Capacity horizon [TU]	–	{20, 26, 56}
Methods to estimate capacity requirement (A_m_)	–	2 methods
Methods to calculate unbounded capacity provided (E_m_)	–	2 methods
Capacity flexibility	–	{0.1, 0.3}
WIP-cap-change-ratio	–	{0, 0.25, 1.5}
Parameter combinations	480	959 616
Replications	10	10
Simulation runs	4 800	9 596 160

## Numerical results

To comprehensively explore the potential improvements offered by ConWIP extensions and their impact on production system behaviour, i.e. optimal planning parameters, we undertake a systematic investigation. Initially, we present the system performance by applying the workload-based ConWIP production planning and control method. Subsequently, we concentrate on the impact of integrating flexible capacity, followed by an analysis of two capacity backward scheduling approaches and the integration of safety lead time. Finally, we comprehensively examine all the presented ConWIP extensions, including the effects of dynamic WIP-Cap, to gain a holistic understanding of their contributions.

### Workload-based ConWIP

**Fig. 4:**
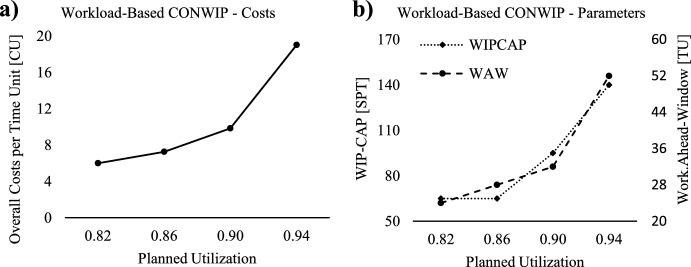
Lowest overall costs and optimal planning parameters for workload-based ConWIP

As described in Sect. 4*Simulation* *Model* *and* *Numerical* *Study*, for the workload-based ConWIP an enumeration of the planning parameters *WIP-Cap* and *Work-Ahead-Window* is conducted to identify the optimal planning parameters and the respective costs. Unlike Thürer et al. (2019), our analysis does not extend to comparing our results with conventional ConWIP or the different load measurements developed by Oosterman et al. (2000), as their research has already demonstrated that this connection to shop load with WIP-Cap is the most effective. Instead, our investigation centres on the influence of planned utilization on performance and the optimal parameterization of WIP-Cap, providing decision-makers with valuable insights to understand the impact of planned utilization adjustments on operational efficiency. Therefore, Fig. 4a presents the lowest costs per time unit [TU] for each planned utilization scenario, Fig. 4b showcases the respective optimal *WIP-Cap*, measured in standard processing time [SPT], and the optimal *Work-Ahead-Window*, measured in time units [TU].

The results imply that a higher planned utilization necessitates an increase in both the WIP-Cap and Work-Ahead-Window. Consequently, more production orders need to be released earlier into the production system to offset the increased workload, leading to higher overall costs due to increased WIP and FGI. Additionally, tardiness costs rise as the production system operates at a higher load.

### Effect of flexible capacity

**Fig. 5:**
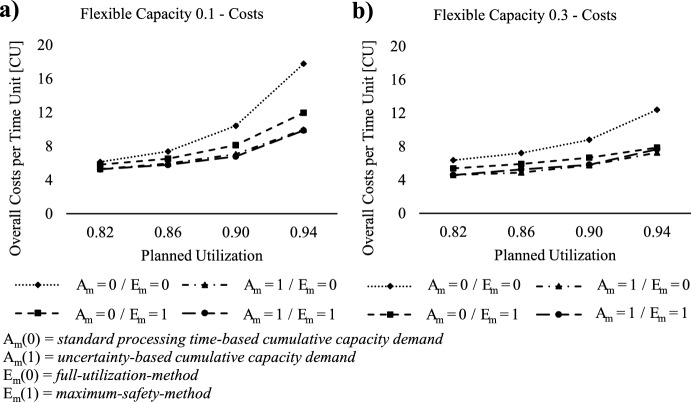
Lowest overall costs for flexible capacity integration into ConWIP

To identify the effect of flexible capacity within ConWIP, the methods from Altendorfer et al. (2014) with their respective extensions as introduced in Sect. 3.2*ConWIP* *Extensions* are applied here without any adaptions to the workload-based ConWIP. This means, that the *processing* *time-based* *backward* *scheduling* from Eq. (3) is applied with safety lead time *S* = 0 and *WIP-Cap-Change-Ratio* *R* = 0. While, Altendorfer et al. (2014) investigated the capacity setting methods for a queuing model, without applying a production planning and control method, we now apply workload-based ConWIP. Figure 5a displays the lowest overall costs for capacity flexibility = 0.1, and Fig. 5b presents the lowest overall costs for capacity flexibility = 0.3, both for all four different combinations of $A_{m} \left( \cdot \right)$ and $E_{m} \left( \cdot \right)$. In relation to $A_{m} \left( \cdot \right)$, the notation "0" represents the *standard* *processing* *time-based* *cumulative* *capacity* *requirement*, while "1" denotes the *uncertainty-based* *cumulative* *capacity* *requirement*. Regarding $E_{m} \left( \cdot \right)$, "0" indicates the *full-utilization-method*, while "1" represents the *maximum-safety-method*.

Comparing both figures, a higher capacity flexibility results in lower overall costs for all four different combinations of $A_{m} \left( \cdot \right)$ and $E_{m} \left( \cdot \right)$. Additionally, increased capacity flexibility leads to less overall cost escalation at higher planned utilization scenarios, as the production system gains greater adaptability to workload peaks. Regarding the comparison of the four capacity setting methods of $A_{m} \left( \cdot \right)$ and $E_{m} \left( \cdot \right)$, the results indicate the necessity of integrating safety capacity into the capacity setting. Specifically, the combination of *standard* *processing* *time-based* *cumulative* *capacity* *requirement* (*A*_*m*_ = 0) and *maximum-safety-method* (*E*_*m*_ = 0) leads to significantly higher costs for both investigated levels of capacity flexibility across all planned utilization scenarios. Moreover, both figures demonstrate that incorporating safety capacity during the estimation of capacity requirement results in lower overall costs across all planned utilization scenarios than integrating it during the calculation of the unbounded capacity provided. Consequently, applying *uncertainty-based* *cumulative* *capacity* *requirement* (*A*_*m*_ = 1) combined with *full-utilization-method* (*E*_*m*_ = 0) yields lower overall costs compared to the combination of *standard* *processing* *time-based* *cumulative* *capacity* *requirement* (*A*_*m*_ = 0) and *maximum-safety-method* (*E*_*m*_ = 1). The integration of safety capacity at both, the estimation of capacity requirement and the calculation of the unbounded capacity provided, does not significantly alter the system performance. Slightly lower overall costs occur for the combination of *uncertainty-based* *cumulative* *capacity* *requirement* (*A*_*m*_ = 1) and *maximum-safety-method* (*E*_*m*_ = 1) at capacity flexibility = 0.1. However, at capacity flexibility = 0.3, the costs increase compared to the combination of *uncertainty-based* *cumulative* *capacity* *requirement* (*A*_*m*_ = 1) and *full-utilization-method* (*E*_*m*_ = 0). These observations closely align with the basic scenario analysed by Altendorfer et al. (2014), where the application of safety capacity at both, the estimation of capacity requirements and during the calculation of unbounded capacity provision, led to performance enhancements. However, unlike the findings in Altendorfer et al. (2014), the application of ConWIP demonstrates a higher performance increase when capacity flexibility increases, a phenomenon not observed in their study. We relate this performance enhancement to the restricted WIP within the production system, which is based on the ConWIP logic.

To conduct a comprehensive investigation into the impact of flexible capacity, Table 2 provides a detailed comparison between the workload-based ConWIP (WB) and the extended ConWIP by capacity, considering both investigated levels of capacity flexibility. This table presents the lowest overall costs [CU] with the respective confidence interval for *α* = 0.01, the corresponding planning parameters and the observed production KPIs for each planned utilization scenario. The KPI: production lead time is the duration from the start of production to the completion of the final product, while the KPI: FGI lead time refers to the time from when a product is completed and enters inventory to when it is delivered to the customer.

**Table 2:** Lowest overall costs and optimal planning parameters for workload-based and capacity extended ConWIP

Planned utilization scenario	0.82			0.86			0.90			0.94		
ConWIP	WB	Flex.=0.1	Flex.=0.3	WB	Flex = 0.1	Flex. =0.3	WB	Flex.=0.1	Flex.=0.3	WB	Flex.=0.1	Flex.=0.3
WIP-Cap [SPT]	65	65	50	65	65	50	95	65	65	140	110	80
Work-Ahead-Window [TU]	24	20	20	28	24	20	32	28	24	52	32	28
Capacity horizon	–	38	26	–	32	26	–	38	26	–	44	32
Estimation capacity requirement (A_m_)	–	1	1	–	1	1	–	1	1	–	1	1
Calculation unbounded capacity provided (E_m_)	–	1	0	–	1	0	–	1	0	–	1	0
WIP costs per time unit [CU]	2.22	2.18	1.88	2.44	2.45	2.01	3.21	2.70	2.56	4.28	3.96	3.27
FGI costs per time unit [CU]	3.08	1.98	2.12	3.83	2.80	1.86	4.10	3.27	2.71	4.94	3.18	2.91
Tardiness costs per time unit [CU]	0.70	1.10	0.57	0.98	0.54	1.00	2.53	0.82	0.48	9.81	2.74	1.07
Total costs per time unit [CU]	**6.00**	**5.27**	**4.57**	**7.26**	**5.78**	**4.86**	**9.84**	**6.78**	**5.74**	**19.03**	**9.89**	**7.25**
Confidence interval ± [CU]	0.20	0.18	0.11	0.34	0.11	0.13	0.89	0.18	0.08	3.85	1.17	1.00
Mean Production lead time [TU]	13.53	13.29	11.48	14.20	14.23	11.69	17.85	14.98	14.20	22.79	21.11	17.42
Mean FGI lead time [TU]	9.43	6.10	6.52	11.20	8.18	5.44	11.43	9.12	7.57	13.19	8.50	7.77
Mean Tardiness [TU]	0.94	1.49	0.76	1.27	0.70	1.28	3.11	1.01	0.59	11.54	3.23	1.26

As observed in Table 2, the integration of capacity flexibility results in lower values for WIP-Cap and Work-Ahead-Window compared to workload-based ConWIP. This effect is more pronounced with higher capacity flexibility, suggesting that the production system manages workload peaks by adjusting capacity instead of releasing more production orders earlier into the system. As a result, WIP and FGI costs decrease across all planned utilization scenarios. The cost differences between WB, Flex. = 0.1 and Flex. = 0.3 are statistically significant with *α* = 0.01 for each observed planned utilization. Moreover, the narrowness of the confidence intervals when capacity flexibility is integrated underscores the statistical significance and reliability of these cost reductions.

The performance enhancement of flexible capacity is also noticeable in the FGI lead time, as higher planned utilization at the workload-based ConWIP results in higher mean FGI lead times, whereas with capacity flexibility = 0.3, only marginal changes are observed. Additionally, the results demonstrate that higher capacity flexibility allows for a reduced Capacity Horizon. Consequently, the production system can handle smaller customer required lead times, as the workload of production orders can be considered for capacity calculations at a later stage, which is a benefit for make-to-order production systems. Upon examining the production lead time, the findings suggest that a higher planned utilization leads to longer mean production lead times, particularly in the case of workload-based ConWIP. This heightened production system load also results in higher mean tardiness for the workload-based ConWIP, as workload peaks cannot be managed by adjusting capacity.

### Effect of safety lead time and different capacity backward scheduling approaches

**Fig. 6:**
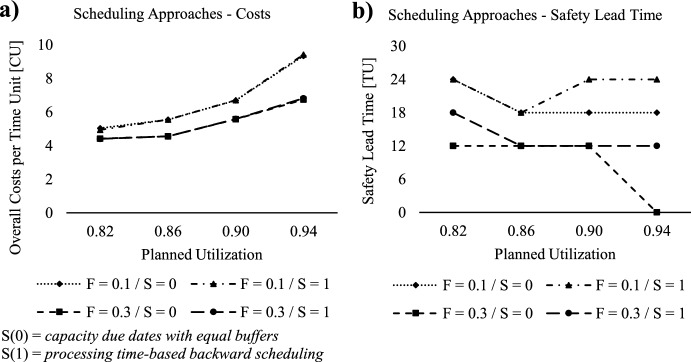
Lowest overall costs and optimal safety lead time for scheduling approaches

In this section, we investigate the performance of the safety lead time, along with the two developed capacity backward scheduling approaches introduced in Sect. 3.2*ConWIP* *Extensions*. Specifically, we explore the *processing* *time-based* *backward* *scheduling* approach and *capacity* *due* *dates* *with* *equal* *buffers* approach, again excluding the WIP-Cap adjustment in this analysis, i.e. *WIP-Cap-Change-Ratio* *R* = 0. This investigation is inspired by Altendorfer et al. (2014), who enhanced production system performance by applying a complex statistical method for calculating capacity due date buffers based on the distribution of the customer-required lead time. In contrast, we aim to refine our results by applying a practically traceable safety lead time. The use of a safety lead time has demonstrated significant performance enhancements in scenarios of uncertainty when applied in Material Requirements Planning (MRP) context, as evidenced by van Kampen et al. (2010) or Hopp and Spearman (2011). However, unlike these studies, which applied safety lead times in the context of generating production plans, i.e. determining plan start and end dates, our application is exclusively focused on scheduling capacity. Figure 6a shows the lowest costs per time unit [TU] for each planned utilization scenario for both scheduling approaches and both investigated levels of capacity flexibility (F). Figure 6b showcases the respective optimal applied safety lead time, measured in time units [TU]. In relation to backward scheduling approaches, the notation "0" signifies the *capacity* *due* *dates* *with* *equal* *buffers*, while "1" denotes the *processing* *time-based* *backward* *scheduling*.

As depicted in Fig. 6a, both capacity backward scheduling approaches yield nearly identical costs for both levels of capacity flexibility. The similarity in outcomes suggests that both methods are effective for scheduling capacity due dates, underscoring their value in improving performance by applying safety lead times. In Fig. 6b, we observe that higher planned utilization results in shorter safety lead times, as the production system must adjust the capacity requirement by scheduling tasks later to cope with the increased workload. Furthermore, the findings suggest that higher capacity flexibility necessitates shorter safety lead times. This is because greater flexibility allows for better adaptation of capacity to compensate for uncertainties in processing time or demand. This observation aligns with van Kampen et al. (2010), who also identified a critical threshold beyond which additional safety lead time ceases to yield performance improvements, however emphasizing its application for production plan generation. Additionally, the results indicate that the *capacity* *due* *dates* *with* *equal* *buffers* approach requires shorter safety lead times at both capacity flexibility levels compared to the *processing* *time-based* *backward* *scheduling*. This can be attributed to the fact that the *processing* *time-based* *backward* *scheduling* approach lacks buffers between individual machines, whereas the *capacity* *due* *dates* *with* *equal* *buffers* approach incorporates buffers at each machine.

### Effect of dynamic WIP-Cap

**Fig. 7:**
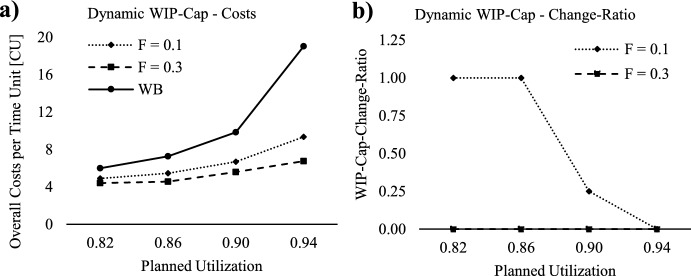
Lowest overall costs and optimal WIP-Cap-Change-Ratio for scheduling approaches

In this Section, we explore the integration of dynamic WIP-Cap to fully leverage the potential of flexible capacity. Figure 7a showcases the lowest costs per time unit [TU] for each planned utilization scenario, considering the extended ConWIP by capacity and WIP-Cap adjustment, across both levels of capacity flexibility (F) that were investigated. For a comprehensive comparison, Fig. 7a also includes the lowest costs per time unit [TU] for the workload-based ConWIP (WB), as discussed in Sect. 5.1*Workload-based* *ConWIP*. Concurrently, Fig. 7b displays the respective optimal *WIP-Cap-Change-Ratio,* for both levels of capacity flexibility (F). In Fig. 7a, it is evident that the extended ConWIP by capacity and WIP-Cap adjustment leads to significantly lower overall costs for both investigated levels of capacity flexibility, regardless of the planned utilization scenario.

Notably, our data indicates that higher capacity flexibility significantly reduces overall costs, a point further emphasized in Fig. 7b, where it becomes clear that having more capacity flexibility makes WIP-Cap adjustments unnecessary. This finding stands in contrast to the research by Belisario et al. (2015) or Azouz and Pierreval (2019), where increased frequency of card changes, i.e., WIP-Cap adjustment, correlates with enhanced performance. Hence, our results suggest that capacity flexibility is more crucial than WIP-Cap adjustment, offering a contrary perspective to these earlier publications by highlighting a different approach to enhancing production system performance. However, when capacity flexibility is set to 0.1, the production system still needs WIP-Cap adjustments, except at the highest planned utilization scenario. This reveals that WIP-Cap adjustment serves as an additional flexibility tool, allowing the production system to adapt to changes in throughput potential triggered by adjustments in realized capacity. Unlike other dynamic card controlling methods, such as those by Hopp and Roof (1998), Belisario et al. (2015) or Azouz and Pierreval (2019), our approach to WIP-Cap adaptation is executed periodically, rather than in response to stochastic events like customer order arrivals or threshold breaches, thus minimizing the nervousness within the production system.

In order to comprehensively analyze the overall results and assess the impact of the ConWIP extensions, Table 3 offers an in-depth comparison between the workload-based ConWIP (WB) and the extended ConWIP by capacity and WIP-Cap adjustment, considering both investigated levels of capacity flexibility. This table presents the lowest overall costs [CU] with the respective confidence interval for *α* = 0.01, the corresponding planning parameters, and the respective production KPIs for each planned utilization scenario.

**Table 3:** Lowest overall costs and optimal planning parameters for workload-based and extended ConWIP

Planned utilization scenario	0.82			0.86			0.90			0.94		
ConWIP	WB	Flex. =0.1	Flex.=0.3	WB	Flex.=0.1	Flex.=0.3	WB	Flex.=0.1	Flex.=0.3	WB	Flex.=0.1	Flex.=0.3
WIP-Cap [SPT]	65	50	50	65	50	50	95	65	65	140	110	65
Work-Ahead-Window [TU]	24	20	20	28	24	20	32	24	24	52	32	28
Capacity horizon	–	44	38	–	32	32	–	32	32	–	32	32
Estimation capacity requirement (A_m_)	–	1	1	–	1	1	–	1	1	–	1	0
Calculation unbounded capacity provided (E_m_)	–	0	0	–	0	0	–	0	0	–	0	1
Capacity backward scheduling approach	–	1	1	–	1	1	–	0	0	–	0	0
Safety lead time [TU]	–	24	18	–	18	12	–	12	12	–	18	0
WIP-cap-change-ratio	–	1.00	0	–	1.00	0	–	0.25	0	–	0	0
WIP costs per time unit [CU]	2.22	1.98	1.88	2.44	2.13	2.01	3.21	2.72	2.55	4.28	3.98	2.71
FGI costs per time unit [CU]	3.08	1.95	2.19	3.83	2.58	1.93	4.10	2.18	2.80	4.94	3.15	3.47
Tardiness costs per time unit [CU]	0.70	0.96	0.34	0.98	0.74	0.62	2.53	1.78	0.23	9.81	2.22	0.57
Total costs per time unit [CU]	**6.00**	**4.89**	**4.40**	**7.26**	**5.45**	**4.56**	**9.84**	**6.68**	**5.58**	**19.03**	**9.34**	**6.75**
Confidence interval ± [CU]	0.20	0.19	0.06	0.34	0.13	0.07	0.89	0.40	0.05	3.85	0.68	0.12
Mean Production lead time [TU]	13.53	12.04	11.44	14.20	12.38	11.68	17.85	15.09	14.16	22.79	21.18	14.42
Service level	0.96	0.93	0.96	0.96	0.95	0.94	0.92	0.90	0.97	0.82	0.91	0.96
Mean Tardiness [TU]	0.94	1.30	0.46	1.27	0.95	0.80	3.11	2.19	0.28	11.54	2.61	0.67

As shown in Table 3, the ConWIP extensions significantly enhance the system performance across all observed planned utilization scenarios. With a capacity flexibility = 0.1, the implementation yields remarkable overall cost reductions with increasing planned utilization of 18, 25, 32, and 51%. Similarly, for a capacity flexibility = 0.3, the overall cost reduction for increasing planned utilization is even more impressive, reaching 27%, 37%, 43%, and 63%. The cost differences between WB, Flex. = 0.1 and Flex. = 0.3 are again statistically significant with *α* = 0.01 for each observed planned utilization. Upon comparison with Table 2, where only flexible capacity is integrated without safety lead time and WIP-Cap-adjustment, three noteworthy distinctions in optimal planning parameters emerge. Firstly, the increased narrowness of the confidence intervals in the highest planned utilization scenario suggests enhanced performance stability, a positive outcome directly linked to employing safety lead time, since no WIP-Cap adjustment is performed. Secondly, when a dynamic WIP-Cap is applied, the WIP-Cap value is lower as it becomes adaptable in response to changes in throughput potential. Thirdly, the introduction of a safety lead time has a significant effect on the required Capacity Horizon, since their interplay influences capacity scheduling.

Moreover, the comparative analysis of the two tables reveals a slight further improvement potential in overall cost and across almost all production KPIs, regardless of the observed planned utilization scenarios. However, the results also show that the highest cost reduction potential is based on the flexible capacity setting which underlines its high practical relevance.

## Conclusion

In this paper, several extensions for a workload-based ConWIP make-to-order multi-stage production system with stochastic demand, processing, and customer required lead times are developed. At first, we extended the workload-based ConWIP by integrating system load-dependent capacity setting methods introduced in Altendorfer et al. (2014). We explore two different methods for estimating required capacity and calculating unbounded capacity, resulting in four combinations of periodical capacity setting methods. To fully capitalize on the benefits of flexible capacity and enable the production system to adapt to changes in throughput potential, we further extended ConWIP by applying a WIP-Cap-Change-Ratio based on the provided capacity. Additionally, we develop two capacity due date scheduling approaches based on the implementation of a safety lead time to optimize system performance.

Employing agent-based discrete-event simulation, we evaluate the performance of the ConWIP extensions through a numerical study encompassing a broad parameter enumeration. The results reveal that integrating flexible capacity significantly enhances the system's performance at both investigated levels of capacity flexibility. This improvement potential is further augmented by implementing a safety lead time, and both developed capacity backward scheduling approaches yield similar results. For the lower level of capacity flexibility, the integration of dynamic WIP-Cap enhances the system's performance even further. However, for the higher level of capacity flexibility, no additional improvement potential is observed when applying a dynamic WIP-Cap. Previous WIP-Cap adjustment literature finds a positive correlation between frequency of card changes and respective performance, but the numerical results in this paper show that no adjustments are needed for high capacity flexibility. Therefore, we conclude that capacity adaptation carries greater significance than dynamic WIP-Cap adjustment. Overall, the improvement potential in terms of overall costs (including WIP, FGI, and tardiness costs) reaches up to 51% or 63% depending on the level of capacity flexibility, compared to workload-based ConWIP.

Additional noteworthy findings include: (1) The ConWIP extension leads to lower optimal planning parameters for both WIP-Cap and Work-Ahead-Window, i.e. the production system is more lean in the sense of inventory and lead time, (2) Higher capacity flexibility allows for a reduced Capacity Horizon, benefiting make-to-order production systems as it allows for considering production orders in capacity calculations at a later stage, i.e. the system is better capable of handling short term orders, and (3) When a dynamic WIP-Cap is applied, the WIP-Cap value is lower as it becomes adaptable in response to changes in throughput potential.

Future research should focus on exploring the applicability of the ConWIP extensions in more complex production systems, such as job shop production systems. These investigations may reveal the necessity of a dynamic WIP-Cap even for higher levels of capacity flexibility.

## Abbreviations

*M*: Set of machines in the production system
*I*: Set of production orders
*∆*: Repeat period
*t*: Auxiliary variable for time
*t*_c_: Current simulation time
*δ*: Capacity horizon
*E[P*_*m*__,__*i*_]: Standard processing time, i.e. expected value, for production order *i* at machine *m*
*P*_*m,i*_: Stochastic realization of processing time for production order *i* at machine *m*
*D*_*i*_: Customer order due date of production order *i*
*O*_*i*_: Production order due date of customer order *i*
*OR*_*i*_: Earliest possible production order release date of production order *i* based on the Work-Ahead-Window
*S*: Safety lead time
*CD*_*m,i*_*(P*_*i*_*)*: Capacity due date at machine *m* for production order *i* at processing time-based backward scheduling method
*CD*_*m,i*_*(OR*_*i*_*)*: Capacity due date at machine *m* for production order *i* at capacity due dates with equal buffers method
*CD*_*m,max*_: Last considered capacity due date within Capacity Horizon *δ* at machine *m*
*A*_*m*_*(t,E[P*_*m*_*,.])*: Standard processing time based cumulative capacity requirement for machine *m* for time *t*
*A*_*m*_*(t,P*_*m*__*,.*_*)*: Uncertainty based cumulative capacity requirement for machine *m* for time *t*
F^−^^1^: Inverse of the CDF (cumulated distribution function)
*β*: Used probability for inverse of the cumulated distribution function (safety value)
*E*_*m*_*(t*_*c*_*,A*_*m*_*(CD*_*m,max*__*,.*_*))*: Unbounded capacity to be provided for each period at full-utilization-method for machine *m* at simulation time *t*_*c*_
*E*_*m*_*(t*_*c*_*,max(.))*: Unbounded capacity to be provided for each period at maximum-safety-method for machine *m* at simulation time *t*_*c*_
*E(t*_*c*_*)*: Maximum of the calculated unbounded capacity of all machines *m* at simulation time *t*_*c*_
*C*_*min*_: Lower limit of the realizable capacity within a *repeat* *period* *∆*
C_*norm*_: Normal realized capacity within a *repeat* *period* *∆*
*C*_*max*_: Upper limit of the realizable capacity within a *repeat* *period*
*C(t*_*c*_*,.)*: Actual realized capacity within a *repeat* *period* *∆* (for every machine *m* in the production system)
*WTA*: Working-time-account
*WTA*_*max*_: Upper limit of the working-time-account
*W*_*norm*_: Determined WIP-Cap for *normal* *provided* *capacity*
*W*_*c*_: Adjusted WIP-Cap (based on *actual* *realized* *capacity* and *WIP-Cap-Change-Ratio*)
*R*: WIP-Cap-Change-Ratio

## Declarations

### Data availability

The data that support the findings of this study are available in Zenodo at 10.5281/zenodo.8228404 upon reasonable request to the author Balwin Bokor.
